# A dynamical traffic flow model for a cognitive drivers' sensitivity in Lagrangian scope

**DOI:** 10.1038/s41598-022-22412-9

**Published:** 2022-10-15

**Authors:** Md. Anowar Hossain, Jun Tanimoto

**Affiliations:** 1 Interdisciplinary Graduate School of Engineering Sciences, Kyushu University, Kasuga-koen, Kasuga-shi, Fukuoka, 816-8580 Japan; 2 Faculty of Engineering Sciences, Kyushu University, Kasuga-koen, Kasuga-shi, Fukuoka, 816-8580 Japan

**Keywords:** Applied mathematics, Computational science

## Abstract

A new microscopic traffic flow model is established based on heterogeneous driver's sensitivity; in this new model, the driver's sensitivity is defined as being dependent on the headway distances to the preceding vehicle, similar to Bando’s optimal velocity function. We introduce the formulation of this cognitive driver's sensitivity utilizing a modified form of Bando’s optimal velocity function. A simple methodology, which is used for improving Bando’s optimal velocity function, has been implemented for developing the cognitive driver’s sensitivity function, which establishes a correlation between the flow field’s density and human drivers' responses. The model is highly advanced for introducing a human-driven traffic flow field considering the driver’s mental behavioral activity. Using the linear stability condition, we elucidate a neutral stability condition. A series of numerical simulations indicates how the present model describes dynamics that differ from the conventional model, which assumes a constant driver's sensitivity.

## Introduction

### Research organization and literature reviews

The complexity of the sciences behind communication physics has been emerging due to various phenomena, including very trivial incidents such as driver’s behavior, prior vehicle’s taillight effect, lane sharing attitude of next door, route facilities, etc. Conventional traffic flow models have also been under development by applying communication physics to specific fundamental events occurring on roads. This is because the complexity of science exponentially increases when increasing model parameters relating to incidents occurring on roads. Moreover, human drivers are not able to respond to all the events that take place on the road, which should be considered for modeling, and this handicap also increases the complexity of theoretical modeling. So far, none of the traffic models can fully describe an actual traffic flow field, considering all the events that take place on the road. Hence, researchers have been trying to implement more detailed traffic flow field models, reducing the gap between the realistic transport system and its current theoretical transport-physics based model. However, a crucial drawback in existing theoretical traffic flow models has been noticed due to the assumption of a constant driver’s sensitivity, which means a unique driver’s attention that is not realistic for a human driver. Unfortunately, this limitation has been assumed for over two decades. Several traffic models, such as Cellular automata models^1–6^ Microscopic models,^7–12^ Lattice hydrodynamic models,^13–17^ and Continuum models,^18–28^ have been published as an extension of conventional traffic models, such as Bando’s optimal velocity (hereafter called OV) model and the full velocity difference (FVD ) model, but this drawback still remains unchanged. Zhipeng Li et al. introduced an excellent traffic model^29^ considering a heterogeneous driver’s sensitivity for each driver, which is a distinguished but time-constant driver’s sensitivity. But the driver’s attention never be fixed, and the flow field’s conditions influence it. This research aims to overcome this limitation in existing by introducing a new cognitive driver’s sensitivity that is highly efficient in tracing the mental behavior of the driver following its headway distances. To conduct this research in a proper direction, these recently published traffic models^30–33^ are highly informative for us, where the authors utilized the three-phase theory to explore some new phenomena, such as velocity feedback strategy, a bottleneck for accident and on-ramp conditions, and critical characteristics of highway traffic flow.

### Research motivation

After this early history of traffic flow research, several commendable traffic models have been developed. Junta Matsukidaira et al.^20^ developed a famous traffic model so-called cellular automaton model for traffic flow systems, but the model doesn’t take into account the driver’s attentiveness which is time constant and the same for all drivers. A model^19^ relating to the spatiotemporal structure of a traffic flow system with an open boundary has been improved by Namiko Mitarai and Hiizu Nakaknishi, where they discovered a new traffic zone, namely “convectively unstable region” beside of stable, metastable, and unstable regions following unique driver’s sensitivity. Lee et al.^27^ proposed a traffic model focusing on the synchronizing flow phase and demonstrated a relationship between mechanical restriction and human overreaction for triggering a congested traffic state. Zhai et al.^34^ proposed an extended car-following model considering the driver's self-anticipation effect on the vehicle's speed. Furthermore, other modified traffic models^29,35–37^ have been established following the driver's characteristics, style, and reactions, such as driving aggressiveness, to describe the human driver's behavior in the microscopic flow system. An external heterogeneous driver's sensitivity model, where every driver is assigned an individual sensitivity value, has been improved by Ossen et al.^38^. Schultz et al.^39^ showed that the driver's sensitivity in microscopic traffic flow system obeys log-normal and normal distribution, and Makridis et al.^40^ demonstrated how the traffic flow dynamic is affected by the heterogeneity of the vehicle-driver system. In recent years, the traffic flow fields have been explored in many ways and various traffic models were developed^41–49^. All of those models do not take into account the driver’s sensitivity value which is an unavoidable phenomenon for human-driven traffic flow systems. Introducing an actual human-driven traffic flow field would not be possible without considering the effect of the actual driver’s mental behavior, which is frequently changing due to the flow field conditions. But all of those models fail to explain how the behavioral activities of human drivers have been affected by the density of the traffic flow field. Over the last two decades, various admirable traffic models have been improved, but to the best of our knowledge, every model relies on the time-constant driver's sensitivity. Conventional microscopic traffic models have a common feature describing the driver's sensitivity, which is imposed as a homogeneous and time-constant sensitivity for all drivers in the domain considered. However, in actual traffic flow, the driver's sensitivity or "attention" is neither homogeneous nor constant in time. It frequently varies depending on the environment of the traffic flow field. The motivation of this work is to deliver an appropriate and sustainable solution addressing this flaw. Hence, we develop a new microscopic traffic flow model that accounts for the instantaneous sensitivity of the drivers based on the density of the region surrounding the driver. Here, a novel driver sensitivity function is introduced, in which the local sensitivity is assumed to be inversely proportional to the headway distances to the preceding vehicle.

This paper is organized as follows. Section "Methodology of cognitive drivers' sensitivity model" explains the formulation of the proposed model, and the neutral stability condition is presented in section "Neutral stability analysis". Section "Nonlinear analysis" and "Numerical simulations" present the model's nonlinear phenomena and numerical analysis, respectively, process and its results. Finally, section "Conclusion" demonstrates the work with the actual findings.

## Methodology of cognitive drivers' sensitivity model

In earlier traffic flow models, the dynamics of traffic flow were described using the theory of fluid flow dynamics through a macroscopic approach or an Eulerian viewpoint; the first-order continuity equation governed this class of models, and it was called the continuum traffic flow model. In 1955, Lighthill, Whitham, and Richard proposed a continuum traffic flow model called the LWR model^26,50,51^. The governing equation of the LWR macroscopic traffic model is:

1 $$\frac{\partial \rho }{{\partial t}} + \frac{{\partial \left( {\rho v} \right)}}{\partial x} = 0$$

where $v$, $\rho$, $t$, and $x$ represent the velocity, density, time, and space of the traffic flow system, respectively. To make the model closed, the LWR model assumes a velocity–density correlation that implies the flow is always at equilibrium, which is unrealistic.

Payne^52^ proposed a modified higher-order continuum traffic model to overcome this drawback by introducing a relaxation term. The mathematical formulation of this model is:

2 $$\frac{\partial v}{{\partial t}} + v\frac{\partial v}{{\partial x}} = - \frac{\mu }{\rho T}\frac{\partial \rho }{{\partial x}} + \frac{{v_{e} - v}}{T}$$

where $\mu$ and $T$ are the anticipation coefficient and relaxation time, respectively.

In 1995, Bando et al.^7^ established a new traffic model, which added a new dimension to the traffic flow analysis by introducing the Lagrangian viewpoint, or microscopic standpoint; this model was an OV model in which the properties of the flow field are formulated based on the behavior of each individual driver or individual changes in velocity (either accelerations or decelerations). The OV model is the most well-accepted micromodel due to its simplicity and transparency. The mathematical expression of the OV model is:

3 $$\frac{{dv_{n} \left( t \right)}}{dt} = a\left[ {\Delta V\left[ {x_{n} \left( t \right)} \right] - v_{n} \left( t \right)} \right]$$

where $V\left( {\Delta x_{n} \left( t \right)} \right)$ and $v_{n} \left( t \right)$ are the OV velocity and current velocity of vehicle *n* at time $t$, respectively; in this formulation, $a$ denotes the sensitivity of all drivers, which is assumed to be homogeneous, and $\,\Delta x_{n} \left( t \right)$ represents the headway distances for the $n$ th vehicle at time $t$, which is defined by $\Delta x_{n} \left( t \right) = x_{n + 1} \left( t \right) - x_{n} \left( t \right)$.

Following the OV model, there have been many proposed model variants. For example, in 1998, Helbing and Tilch^10^ proposed an updated version of the OV model by introducing a negative speed difference term, which was called the generalized force (GF) model. The mathematical formulation of the GF model is:

4 $$\frac{{dv_{n} \left( t \right)}}{dt} = a\left[ {V\left[ {\Delta x_{n} \left( t \right)} \right] - v_{n} \left( t \right)} \right] + \lambda \Delta v_{n} \left( t \right)H\left[ { - \Delta v_{n} } \right]$$

where $H\left[ * \right]$ is the Heaviside function; $\Delta v_{n} \left( t \right)$ is the speed difference between the $n$ th car and the $n$ + 1th car, which is measured by $\Delta v_{n} \left( t \right) = v_{n + 1} \left( t \right) - v_{n} \left( t \right)$; and $\lambda$ is another sensitivity coefficient that is completely different from $a$.

The addition of the negative velocity difference enhanced the stability of a given traffic flow field; however, in 2001, Jiang et al.^8^ claimed that the positive velocity difference could also play a significant role in enhancing the flow field stability besides the negative velocity. The full velocity difference (FVD) model was then proposed. The governing equation of the FVD model is:

5 $$\frac{{dv_{n} \left( t \right)}}{dt} = a\left[ {V\left[ {\Delta x_{n} \left( t \right)} \right] - v_{n} \left( t \right)} \right] + \lambda \Delta v_{n} \left( t \right)$$

where all symbols used take their previously defined values.

The present model (hereafter referred to as the heading-gap dependent drivers' sensitivity (HDDS) model postulates that each driver's sensitivity varies with the gap to his/her preceding vehicle. The mathematical formulation of the HDDS model is as follows:

6 $$\frac{{dv_{n} \left( t \right)}}{dt} = S\left[ {\Delta x_{n} \left( t \right)} \right] \cdot \left[ {V\left[ {\Delta x_{n} \left( t \right)} \right] - v_{n} \left( t \right)} \right]$$

where $S\left[ {\Delta x_{n} \left( t \right)} \right]$ is the sensitivity of the *n*th driver at time *t*; $V\left[ {\Delta x_{n} \left( t \right)} \right]$ is the OV of the *n*th vehicle; and $v_{n} \left( t \right)$ is the instantaneous velocity of the *n*th vehicle at time *t*.

We assume that each driver's sensitivity is quantified by the following function:

7 $$S\left[ {\Delta x_{n} \left( t \right)} \right] = a_{min} + \frac{{a_{max} - a_{min} }}{{1 + e^{{\left( {\Delta x_{n} \left( t \right) - h_{c} } \right)}} }}$$

where $a_{max}$ and $a_{min}$ are the upper and lower boundaries of the driver's sensitivity range, respectively. These are positive constant values with a range of $0 < a_{min} < 1$ and $1 < a_{max} < 2$, while the average value of $a_{max}$ and $a_{min}$ is equal to 1 (one). More precisely, at *t* time, the *n*th driver's response time delay is quantified by $\tau \left( {\Delta x_{n} \left( t \right)} \right) = \frac{1}{{S\left( {\Delta x_{n} \left( t \right)} \right)}}$ while $\tau_{max} = \frac{1}{{a_{min} }}$, and $\tau_{min} = \frac{1}{{ a_{max} }}$ indicating the maximum and minimum driver's response time delay amount, and are also constant value parameters. The $h_{c}$ is the safety distance between two adjacent vehicles, which is a parameter shared with the OV function.

The sensitivity function, as defined in Eq. (7), intends to capture a driver's response (sensitivity, or say 'attention') affected by the input headway distances information, which is inspired by the OV function as presumed by several precursors^53–55^. Yet, we think an empirical justification would be needed.

The OV function is given by:

8 $$V\left[ {\Delta x_{n} \left( t \right)} \right] = \frac{{v_{{{\text{max}}}} }}{2} \cdot \left[ {{\text{tanh}}\left( {\Delta x_{n} \left( t \right) - h_{c} } \right) + {\text{tanh}}\left( {h_{c} } \right)} \right]$$

where $v_{{{\text{max}}}}$ is the maximum velocity of the vehicles in the free-flow state.

**Figure 1:**
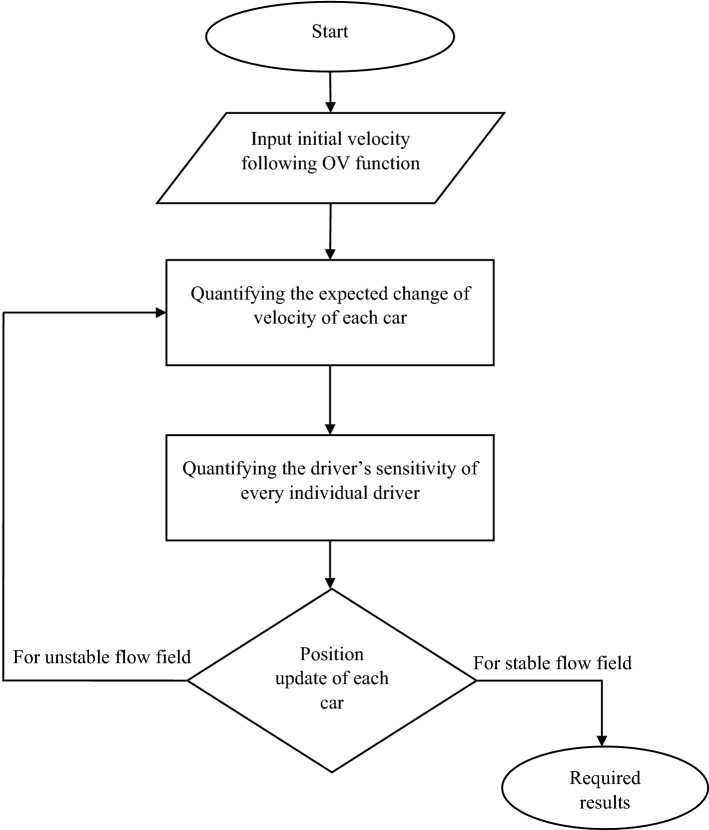
The methodology flow-diagram of the cognitive drivers' sensitivity model.

In terms of the methodology of this work, the physical meaning of Eq. (6) should be confirmed. It is clear that Eq. (6) keeps the form of the so-called OV model, where each of the drivers accelerates/decelerates according to the velocity difference between his ideal velocity (that is dependent on the gap to his preceding vehicle, given by OV function; Eq. 8) and his current one. However, unlike the conventional OV model, the sensitivity, denoted by *S*, is time-variable because it is defined as local-field dependent (more precisely, depending on the gap to his preceding vehicle; Eq. 7). To ensure our model structure, Fig. 1 shows the methodology flow-chart of this model.

**Figure 2:**
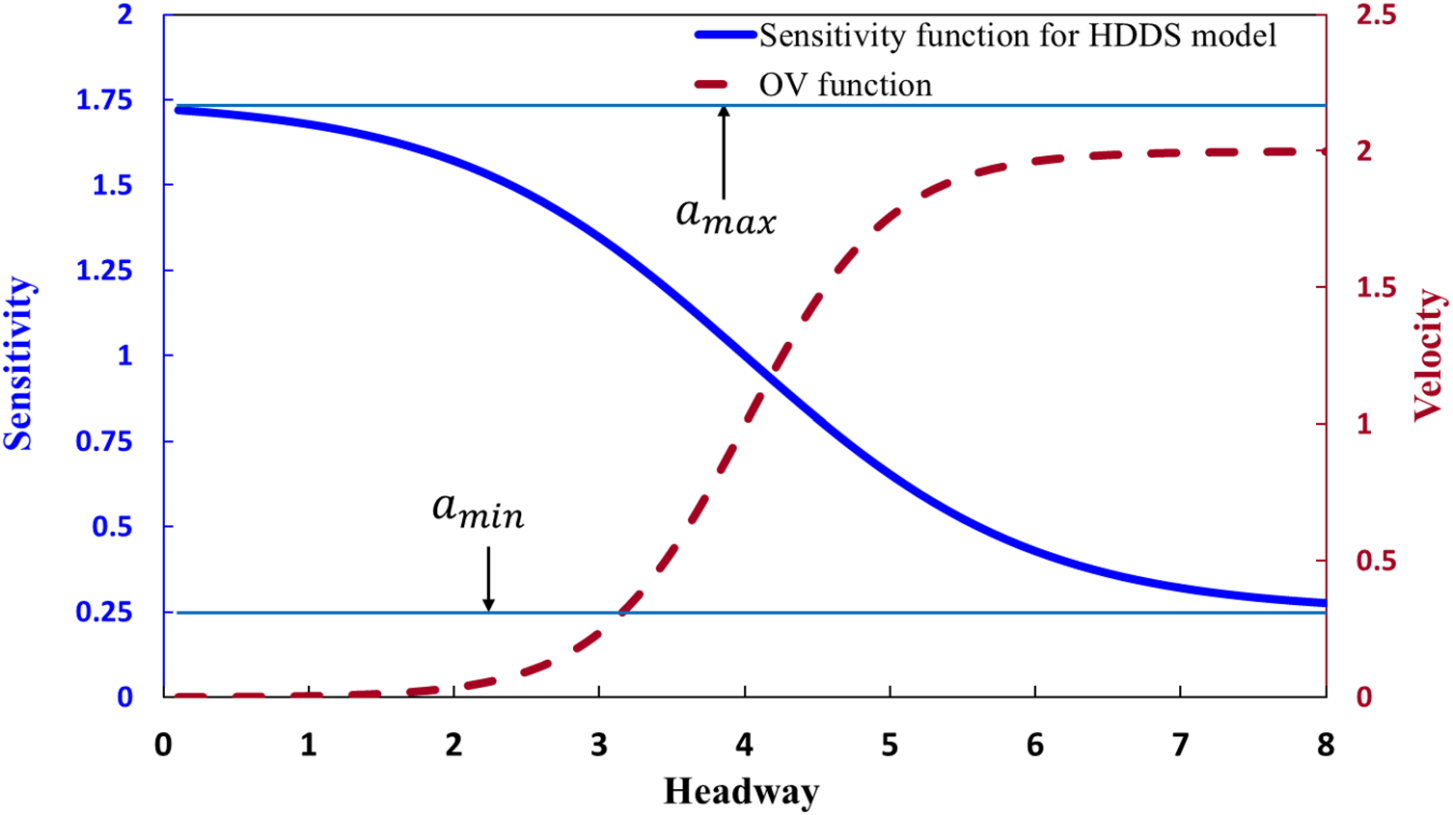
Line graphs for the tendency of sensitivity function (blue line) for HDDS.

Figure 2 shows the possible forms for $S\left[ {\Delta x_{n} \left( t \right)} \right]$ and $V\left[ {\Delta x_{n} \left( t \right)} \right]$ as examples, where the parameters have the values $v_{{{\text{max}}}} = 2$, $a_{max} = 1.75$, and $a_{min} = 0.25$. All of the parameters used in this study are dimensionless.

## Neutral stability analysis

### Mathematical analysis

To assess the performance of the HDDS model, a neutral stability analysis is performed following the linear stability theory. In the model framework, we assume that every vehicle moves with a constant headway distance $b$*.* For this uniform traffic flow, the steady-state solution of Eq. (6) and driver's sensitivity of Eq. (7) are given by^7^:

9 $$\begin{aligned} x_{n}^{0} \left( t \right) &= bn + V\left( b \right)t \\ S_{n}^{0} \left( t \right)& = bn + S\left( b \right)t \\ {\text{with}}\quad b &= L/N \\ \end{aligned}$$

where *L* is the length of the road; and *N* is the total number of vehicles on the entire road.

We assume that $y_{n} \left( t \right)$ and $S_{c} \left( t \right)$ are the small deviation from the steady-state solution $x_{n}^{0} \left( t \right)$ and driver's sensitivity $S_{n}^{0} \left( t \right)$, respectively. Thereafter, the deviation state for the *n*th vehicle is given by:

10 $$\begin{aligned} & x_{n} \left( t \right) = x_{n}^{0} + y_{n} \left( t \right) \\ & S_{n} \left( t \right) = S_{n}^{0} + S_{c} \left( t \right) \\ & {\text{with}}\;\left| {S_{c} \left( t \right)} \right|^{\prime \prime } a_{\max } - a_{\min } \\ \end{aligned}$$

By substituting Eqs. (9) and (10) into Eq. (6), we obtain the simplified form of the model:

11 $$\frac{{d^{2} y_{n} \left( t \right)}}{{dt^{2} }} = \left[ {S\left( b \right) + S^{\prime}\left( b \right)\Delta y_{n} \left( t \right)} \right] \cdot \left[ {V^{\prime}\left( b \right)\Delta y_{n} \left( t \right) - \frac{{dy_{n} \left( t \right)}}{dt}} \right] + \lambda \frac{{d\Delta y_{n} \left( t \right)}}{dt}$$

where $\Delta y_{n} \left( t \right) = y_{n + 1} \left( t \right) - y_{n} \left( t \right)$ and $V^{\prime } \left( b \right) = \frac{{dv\left( {\Delta x_{n} } \right)}}{{d\Delta x_{n} }}_{{\left| {\Delta x_{n} = b} \right.}}$. Expanding $y_{n} \left( t \right) = {\text{exp}}\left( {ikn + zt} \right)$, we obtain the following standard form of the equation:

12 $$z^{2} = \left[ {S\left( b \right) + S^{\prime}\left( b \right)\left( {e^{ik} - 1} \right)e^{ikn + zt} } \right] \cdot \left[ {V^{\prime}\left( b \right)\left( {e^{ik} - 1} \right) - z} \right]$$

Assuming that $z = z_{0} \left( {ik} \right) + z_{1} \left( {ik} \right)^{2} + z_{2} \left( {ik} \right)^{3} + \cdots$ , where $z_{0}$ is a free variable and substituting this into Eq. (12), we obtain the coefficients of the second-and third-order terms of *ik* as follows:

13 $$z_{1} = V^{\prime}\left( b \right)$$

14 $$z_{2} = 0$$

The traffic flow becomes unstable under a small external disturbance force if the following inequality is satisfied:

15 $$a_{max} - a_{min} < \left( {1 + e^{{\left( {\Delta x_{n} \left( t \right) - h_{c} } \right)}} } \right) \cdot \left( {2V^{\prime}\left( b \right) - a_{nim} } \right)$$

Therefore, the neutral stability condition of the new model is given by:

16 $$a_{max} - a_{min} = \left( {1 + e^{{\left( {\Delta x_{n} \left( t \right) - h_{c} } \right)}} } \right) \cdot \left( {2V^{\prime}\left( b \right) - a_{nim} } \right)$$

The linear stability criteria given in Eq. (16) are dependent on the headway distance, and several traffic flow states can be determined based on the following inequalities:

(i) For stable state:
  17 $$a_{max} - a_{min} > \left( {1 + e^{{\left( {\Delta x_{n} \left( t \right) - h_{c} } \right)}} } \right) \cdot \left( {2V^{\prime}\left( b \right) - a_{nim} } \right)$$

(ii) For marginal state:
  18 $$a_{max} - a_{min} = \left( {1 + e^{{\left( {\Delta x_{n} \left( t \right) - h_{c} } \right)}} } \right) \cdot \left( {2V^{\prime}\left( b \right) - a_{nim} } \right)$$
(iii) For unstable state:
  19 $$a_{max} - a_{min} < \left( {1 + e^{{\left( {\Delta x_{n} \left( t \right) - h_{c} } \right)}} } \right) \cdot \left( {2V^{\prime}\left( b \right) - a_{nim} } \right)$$

### Results and discussion of linear stability analysis

**Figure 3:**
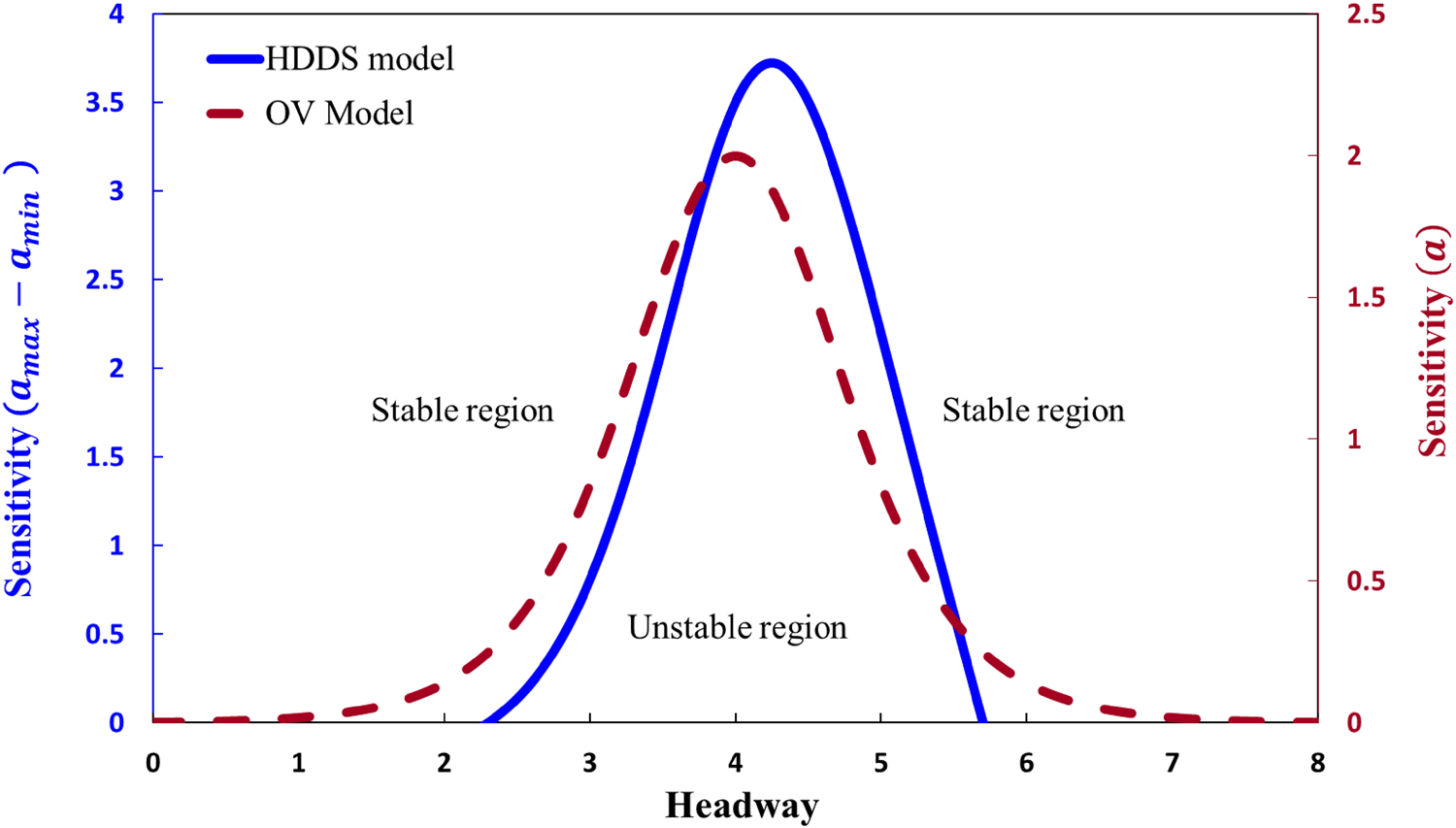
Phase diagram for sensitivity–headway space. The solid blue line indicates the neutral stability history of the HDDS model whereas dashed red line for the conventional OV model.

Figure 3 shows the neutral stability state of the HDDS (blue line) and OV (red line) models. The neutral stability curve of the HDDS model is generated based on the linear stability criteria described by Eq. (16). As defined in Eq. (16), the HDDS model permits each driver's sensitivity to change depending on headway distances, whereas the OV model does not, i.e., the HDDS model does not assume a constant sensitivity. Interestingly, despite this difference, the general tendency resulting from the linear stability analysis for the two different models appears almost similar. In the OV model, under either higher or lower density (i.e., smaller or larger headway distance), a relatively small *a* ensures a stable flow, whereas a relatively large sensitivity is required in the intermediate density region. Similarly, in the HDDS model, a relatively large $a_{max} - a_{min}$ is needed in the intermediate density region, whereas a relatively small $a_{max} - a_{min}$ ensures stability in higher and lower density regions. At a glance, the new model is inferior in the intermediate density region, but the model is highly efficient for the higher or lower density area compared with the OV model.

## Nonlinear analysis

In this section, we deliberate the nonlinear analysis of the proposed HDDS traffic model. To investigate the nonlinear properties of this model, we derived the Korteweg–de Vries (mKdV) equation following kink–antikink wave solution. However, the governing equation of this model, Eq. (6), is transformed into the headway distances form, which is as follows:

20 $$\frac{{d^{2} \Delta x_{n} \left( t \right)}}{{dt^{2} }} = \left[ {S\left[ {\Delta x_{n + 1} \left( t \right)} \right] - S\left[ {\Delta x_{n} \left( t \right)} \right]} \right] \cdot \left[ {V\left[ {\Delta x_{n + 1} \left( t \right)} \right] - V\left[ {\Delta x_{n} \left( t \right)} \right] - \frac{{d\Delta x_{n} \left( t \right)}}{dt}} \right]$$

To obtain the mKdV equation, we introduce a small positive parameter $\xi$ near the critical point ($S_{c}$,$h_{c}$) in the unstable area of traffic flow. We define the slow-scale variables *X* and *T* ^7,56^, which are transformed as follows:

21 $$X = \xi \left( {n + bt} \right)\;{\text{and}}\;T = \xi^{3} t\;{\text{with}}\;0 < \xi \le 1$$

where $n$ and $t$ indicate the space and time variables, respectively. $b$ is a constant that can be quantified. The headway distance $\Delta x_{n} \left( t \right)$ is given by

22 $$\begin{aligned} \Delta x_{n} \left( t \right) = & h_{c} + \xi \beta \left( {X,T} \right) \\ \Delta x_{n + 1} \left( t \right) = & h_{c} + \xi \beta + \xi^{2} \partial_{X} \beta + \frac{{\xi^{3} }}{2}\partial_{X}^{2} \beta + \frac{{\xi^{4} }}{6}\partial_{X}^{3} \beta + \frac{{\xi^{5} }}{24}\partial_{X}^{4} \beta \\ \end{aligned}$$

Therefore we have,

23 $$\begin{aligned} \frac{{d\Delta x_{n} \left( t \right)}}{dt} = & b\xi^{2} \partial_{X} \beta + \xi^{4} \partial_{T} \beta \\ \frac{{d\Delta x_{n + 1} \left( t \right)}}{dt} = & b\xi^{2} \partial_{X} \beta + \xi^{3} b\partial_{X}^{2} \beta + \xi^{4} \left( {\partial_{T} \beta + \frac{b}{2}\partial_{X}^{3} \beta } \right) + \xi^{5} \left( {\partial_{T} \partial_{X} \beta + \frac{b}{6}\partial_{X}^{4} \beta } \right) \\ \frac{{d^{2} \Delta x_{n} \left( t \right)}}{{dt^{2} }} = & b^{2} \xi^{3} \partial_{X}^{2} \beta + 2b\xi^{5} b\partial_{T} \partial_{X} \beta \\ \end{aligned}$$

We have found the following expression of optimal velocity function for $n$th and $\left( {n + 1} \right)$th vehicles by the series expansion:

24 $$\begin{aligned} V\left[ {\Delta x_{n} \left( t \right)} \right] = & V + \xi \beta V^{\prime} + \frac{1}{6}\xi^{3} \beta^{3} V^{\prime\prime\prime} \\ V\left[ {\Delta x_{n + 1} \left( t \right)} \right] = & V + \xi \beta V^{\prime} + \xi^{3} \left( {\frac{1}{6}\beta^{3} V^{\prime\prime\prime} + \frac{1}{2}V^{\prime}\partial_{X}^{2} \beta } \right) \\ & \quad + \xi^{4} \left( {\frac{1}{2}\beta^{2} V^{\prime\prime\prime}\partial_{X} \beta + \frac{1}{6}V^{\prime}\partial_{X}^{3} \beta } \right) + \xi^{5} \left( {\frac{1}{2}\beta V^{\prime\prime\prime}\left( {\partial_{X} \beta } \right)^{2} + \frac{1}{4}\beta^{2} V^{\prime\prime\prime}\partial_{X}^{2} \beta + \frac{1}{24}V^{\prime}\partial_{X}^{4} \beta } \right) \\ \end{aligned}$$

Similarly, a mathematical expression can be obtained of the sensitivity function for $n$ th and $\left( {n + 1} \right)$ th cars, which are as follows:

25 $$\begin{aligned} S\left[ {\Delta x_{n} \left( t \right)} \right] & = \, S + \xi \beta S^{\prime} + \frac{1}{6}\xi^{3} \beta^{3} S^{\prime\prime\prime} \\ S\left[ {\Delta x_{n + 1} \left( t \right)} \right] & = \, S + \xi \beta S^{\prime} + \xi^{3} \left( {\frac{1}{6}\beta^{3} S^{\prime\prime\prime} + \frac{1}{2}S^{\prime}\partial_{X}^{2} \beta } \right) + \xi^{4} \left( {\frac{1}{2}\beta^{2} S^{\prime\prime\prime}\partial_{X} \beta + \frac{1}{6}S^{\prime}\partial_{X}^{3} \beta } \right)\\ & \quad + \xi^{5} \left( {\frac{1}{2}\beta S^{\prime\prime\prime}\left( {\partial_{X} \beta } \right)^{2} + \frac{1}{4}\beta^{2} S^{\prime\prime\prime}\partial_{X}^{2} \beta + \frac{1}{24}S^{\prime}\partial_{X}^{4} \beta } \right) \\ \end{aligned}$$

After that, Eqs. (22)–(25) substitute into Eq. (20) and applying Taylor's expansion up to the seventh-order of $\xi$, a simplified form of the mathematical equation has been obtained as follows:

26 $$\begin{aligned} & \xi^{3} \frac{{b^{2} }}{{S^{\prime}}}\partial_{X}^{2} \beta + \xi^{5} \left( {\frac{2b}{{S^{\prime}}}\partial_{T} \partial_{X} \beta - \frac{1}{2}V^{\prime}\partial_{X}^{2} \beta } \right) + \xi^{6} \left( {\partial_{T} \beta + \frac{1}{6}V^{\prime}\partial_{X}^{3} \beta + \frac{1}{2}V^{\prime\prime\prime}\partial_{X} \beta^{3} } \right) \\&\quad + \xi^{7} \left( {\frac{1}{4}V^{\prime\prime\prime}\frac{{S^{\prime\prime\prime}}}{{S^{\prime}}} \partial_{X}^{2} \beta + \frac{1}{24}V^{^{\prime} } \partial_{X}^{4} \beta + \frac{1}{2}V^{\prime\prime\prime}\partial_{X}^{2} \beta^{3} } \right) \\ \end{aligned}$$

${\text{where }}V^{\prime}\left( b \right) = \frac{{dv\left( {\Delta x_{n} } \right)}}{{d\Delta x_{n} }}\left| {\Delta x_{n} = b} \right.$ and $V^{\prime\prime\prime}\left( b \right) = \frac{{dv\left( {\Delta x_{n} } \right)}}{{d\Delta x_{n} }}\left| {\Delta x_{n} = b} \right.$ Similarly, $S^{\prime}\left( b \right) = \frac{{dS\left( {\Delta x_{n} } \right)}}{{d\Delta x_{n} }}\left| {\Delta x_{n} = b} \right.$ and $S^{\prime\prime\prime}\left( b \right) = \frac{{d^{3} S\left( {\Delta x_{n} } \right)}}{{d\Delta x_{n}^{3} }}\left| {\Delta x_{n} = b} \right.$.

Relating to the critical point $\left( {S_{c} , h_{c} } \right)$, it can be considered that $S_{c} \left( {\Delta x_{n} } \right) = S\left( {\Delta x_{n} } \right) \cdot \left( {1 + \varepsilon^{2} } \right)$ and $b = V^{\prime}$ and Eq. (26) turns into the following form by eliminating the third- and fifth-order terms of $\xi$:

27 $$\xi^{6} \left( {\partial_{T} \beta - g_{1} \partial_{X}^{3} \beta + g_{2} \partial_{X} \beta^{3} } \right) + \xi^{7} \left( {g_{3} \partial_{X}^{2} \beta + g_{4} \partial_{X}^{4} \beta + g_{5} \partial_{X}^{2} \beta^{3} } \right) = 0$$

where the coefficients of $g_{i}$ are defined in Table 1.

**Table 1:** The coefficients ${\varvec{g}}_{{\varvec{i}}}$ of the proposed model.

$g_{1} = \frac{1}{6}S_{c}^{^{\prime}} V^{\prime }$		$g_{2} = \frac{1}{2}S_{c}^{^{\prime}} V^{\prime \prime \prime }$		$g_{3} = \frac{1}{4}S_{c}^{\prime \prime \prime } V^{\prime}$		$g_{4} = \frac{1}{24}S_{c}^{^{\prime}} V^{\prime }$		$g_{5} = \frac{1}{2}S_{c}^{^{\prime}} V^{\prime \prime \prime }$

We obtained the mKdV equation by following a transformation which is as follows:

28 $$\begin{aligned} T = \frac{1}{{g_{1} }}T^{\prime}\quad {\text{and}}\quad \beta = \sqrt {\frac{{g_{1} }}{{g_{2} }}} \beta^{\prime} \\ \end{aligned}$$

Then, we obtained the following standard mKdV equation with a correction term $O\left( \xi \right)$:

29 $$\partial_{{T^{\prime}}} \beta^{\prime} - \partial_{X}^{3} \beta^{\prime} + \partial_{X} \beta^{{\prime}{3}} + \xi M\left[ {\beta^{\prime}} \right] = 0$$

where $$M\left[ {R^{\prime}} \right] = \frac{1}{{g_{1} }}\left[ {g_{3} \partial_{X}^{2} \beta^{\prime} + g_{4} \partial_{X}^{4} \beta^{\prime} + \frac{{g_{1} g_{5} }}{{g_{2} }}\partial_{X}^{2} \beta^{{\prime}{3}} } \right]$$.

The kink–antikink wave solution of the standard mKdV equation can be obtained by eliminating the correction term $O\left( \xi \right)$ from Eq. (29), which is expressed as follows:

30 $$\beta_{0}^{^{\prime}} \left( {X, T^{\prime}} \right) = \sqrt \kappa {\text{tanh}}\left[ {\sqrt {\frac{\kappa }{2}} \left( {X - \kappa T^{\prime}} \right)} \right]$$

The following solvability condition^7^ must be satisfied by Eq. (30) to obtain the value of propagation velocity $\kappa$ for the kink–antikink solution of this mKdV equation:

31 $$\left( {\beta_{0}^{^{\prime}} , M\left[ {\beta^{\prime}} \right]} \right) = \mathop \smallint \limits_{ - \infty }^{\infty } dX\beta_{0}^{^{\prime}} \cdot M\left[ {\beta_{0}^{^{\prime}} } \right] = 0$$

where $M\left[ {\beta_{0}^{^{\prime}} } \right] = M\left[ {\beta^{\prime}} \right]$. We obtained the following form of selective velocity $\kappa$ by performing the integration over Eq. (31):

32 $$\kappa = \frac{{5g_{2} g_{3} }}{{2g_{2} g_{4} - 3g_{1} g_{5} }}$$

Thus, the kink–antikink wave solution of the mKdV equation is given by the following form:

33 $$\beta \left( {X, T} \right) = \sqrt {\frac{{g_{1} \kappa }}{{g_{2} }}} {\text{tanh}}\sqrt {\frac{\kappa }{2}} \left( {X - \kappa g_{1} T} \right)$$

Therefore, the general form of the kink–antikink wave solution in the headway distance is given as follows:

34 $$\Delta x_{n} = h_{c} + \sqrt {\frac{{g_{1} \kappa }}{{g_{2} }}\left( {\frac{{S_{c} }}{{S_{n - 1} }} - 1} \right)} \tanh \sqrt {\frac{\kappa }{2}\left( {\frac{{S_{c} }}{{S_{n - 1} }} - 1} \right)} \left[ {n + \left( {1 - \kappa g_{1} \left( {\frac{{S_{c} }}{{S_{n - 1} }} - 1} \right)t} \right)} \right]$$

Here, the amplitude *A* of the kink–antikink wave solution is given as follows:

35 $$A = \sqrt {\frac{{g_{1} \kappa }}{{g_{2} }}\left( {\frac{{S_{c} }}{{S_{n - 1} }} - 1} \right)}$$

The kink–antikink wave solution is described by the coexisting phase, which is as follows: $\Delta x_{n} = h_{c} \pm A$, where the free-flow phase is quantified by $\Delta x_{n} = h_{c} + A$ and the congested flow phase is identified by $\Delta x_{n} = h_{c} - A$.

## Numerical simulations

### Initial settings for simulation

To investigate the performance of the developed HDDS traffic model, we carried out a numerical analysis^16^ in this section. For performing the numerical simulation, we derive the discretized form of Eq. (6), which is as follows:

36 $$\begin{aligned} & \Delta x_{n} \left( {t + 2\Delta t} \right) - 2\Delta x_{n} \left( {t + \Delta t} \right) + \Delta x_{n} \left( t \right) = \left[ {S\left[ {\Delta x_{n + 1} \left( t \right)} \right] - S\left[ {\Delta x_{n} \left( t \right)} \right]} \right] \\ & \quad \cdot \left[ {\Delta t^{2} \left[ {V\left[ {\Delta x_{n + 1} \left( t \right)} \right] - V\left[ {\Delta x_{n} \left( t \right)} \right]} \right] - \Delta t\left[ {\Delta x_{n} \left( {t + \Delta t} \right) - \Delta x_{n} \left( t \right)} \right]} \right] \\ \end{aligned}$$

For the numerical simulation, we use cyclic boundary conditions with the following initial conditions:

37 $$\begin{aligned} \Delta x_{n} \left( 0 \right) &= \Delta x_{n} \left( 1 \right) = \Delta x_{0} = L/N, {\text{for}} n \ne \frac{N}{2}, \frac{N}{2} + 1, \\ \Delta x_{n} \left( 0 \right) &= \Delta x_{0} + 0.05, {\text{for}} n = \frac{N}{2} \\ \Delta x_{n} \left( 0 \right) &= \Delta x_{0} - 0.05,{\text{for}} n = \frac{N}{2} + 1 \\ \end{aligned}$$

We used the following dimensionless parameters: the total number of the vehicles, $N$ = 100; the length of the domain, $L$ = 2000; safety distances, $h_{c}$ = 2; and $b = L/N$.

### Results and discussion of numerical simulation

**Figure 4:**
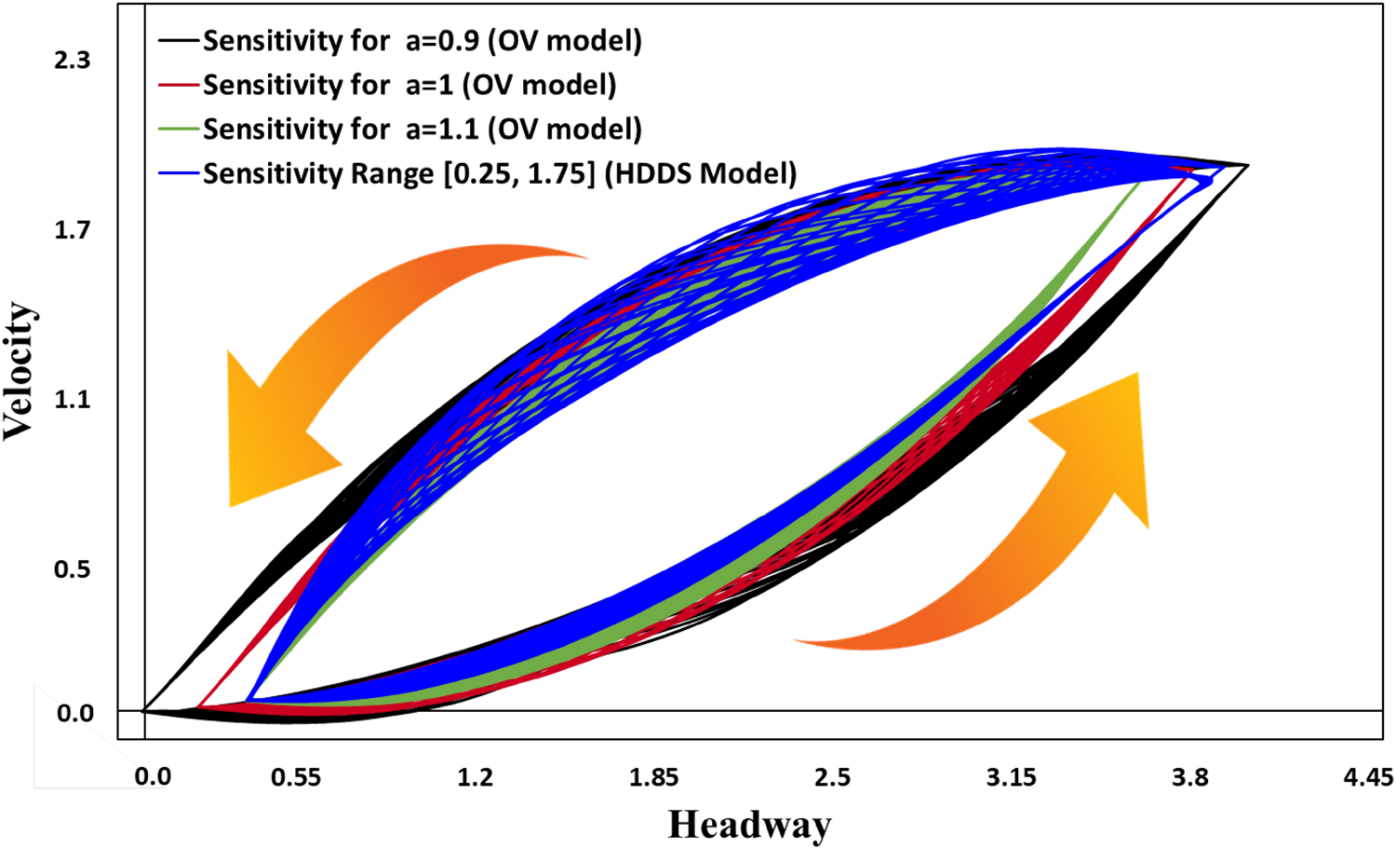
Hysteresis loops for the HDDS model with sensitivity range [0.25, 1.75] and for the conventional OV model with several sensitivities: $a =$ 0.90, 1.00, and 1.10. The upward and downward directed arrows depict the velocity increasing and decreasing tendency respectively.

Figure 4 shows the hysteresis loops of all vehicles in the domain. These hysteresis loops are obtained between the 99900th and 100000th timesteps. In each color depiction, there are 100 loops, because we drew a loop at every timestep. The blue line represents the HDDS model with the assumption $\left[ {a_{min} , a_{max} } \right] = { }$[0.25, 1.75]. This should be primarily comparable with the result in red, which is the case of the OV model assuming $a =$ 1; the similarity of these two models is expected as the average of $a_{min}$ and $a_{max}$ is consistent with the constant sensitivity in the latter case. For comparison, we also show results for $a = { }$ 0.9 (black) and $a =$ 1.1 (green) from the OV model. Inspecting the bottom vertex, we note that the minimal headway distance of the HDDS model (blue) is larger than that of the OV model (red). The HDDS model shows a slightly larger minimum velocity than the OV model. This clearly indicates that the HDDS model delivers a more robust driving situation in higher local densities than the OV model by avoiding markedly lower velocities and extremely small headway distances. This can be explained as being a result of the HDDS model compelling the drivers to behave more sensitively in conditions of minimal headway distance (highest local density) compared with the behavior at larger headway distances (lower local density). Yet, such a feature of variable sensitivity in the HDDS model compared with the OV model imposes the opposing situation at the upper vertex, i.e., at maximal headway distance and maximal velocity, which shows slightly larger headway distance and slightly higher velocity than those obtained in the OV model. This is because the less sensitive driving manner in the HDDS model inevitably brings some sort of inertial effect, leading to larger velocities and headway distances.

The lower and upper bends in the hysteresis loop respectively exhibit the process of velocity increasing, where a vehicle released from a congested cluster resumes to accelerate, and the process of velocity decreasing, where a vehicle with a reasonable speed participates to the tail of a congested jam cluster. Interestingly, those two parts in the hysteresis curve in HDDS model (blue line) are quite different from the conventional OV model (black, red, and green lines).

In , the lower bend shows a relatively lower deviation while the upper bend shows a wider deviation in Y-direction. In contrast, the OV model does not show such a tendency which can be justified as follows. The traffic flow is implemented as a perturbation as defined in Eq. (37). In the upper bend, of which the trajectory starts from maximal velocity (top vertex), each vehicle gradually decelerates from higher to lower speed. Because of this, in the HDDS model, the effect of dynamic sensitivity believers a wider deviation of velocity that originated from the slight perturbation above. On the other hand, in the lower bend, where each vehicle gradually accelerates from the situation of being trapped by a jam cluster (bottom vertex), there is relatively less deviation vis-à-vis than in the upper bend since a vehicle starts from zero velocity. Furthermore, in the conventional OV model, such difference in upper and lower bends in the hysteresis loop does not appear due to the sensitivity; *a*, being kept at a time-constant value.

**Figure 5:**
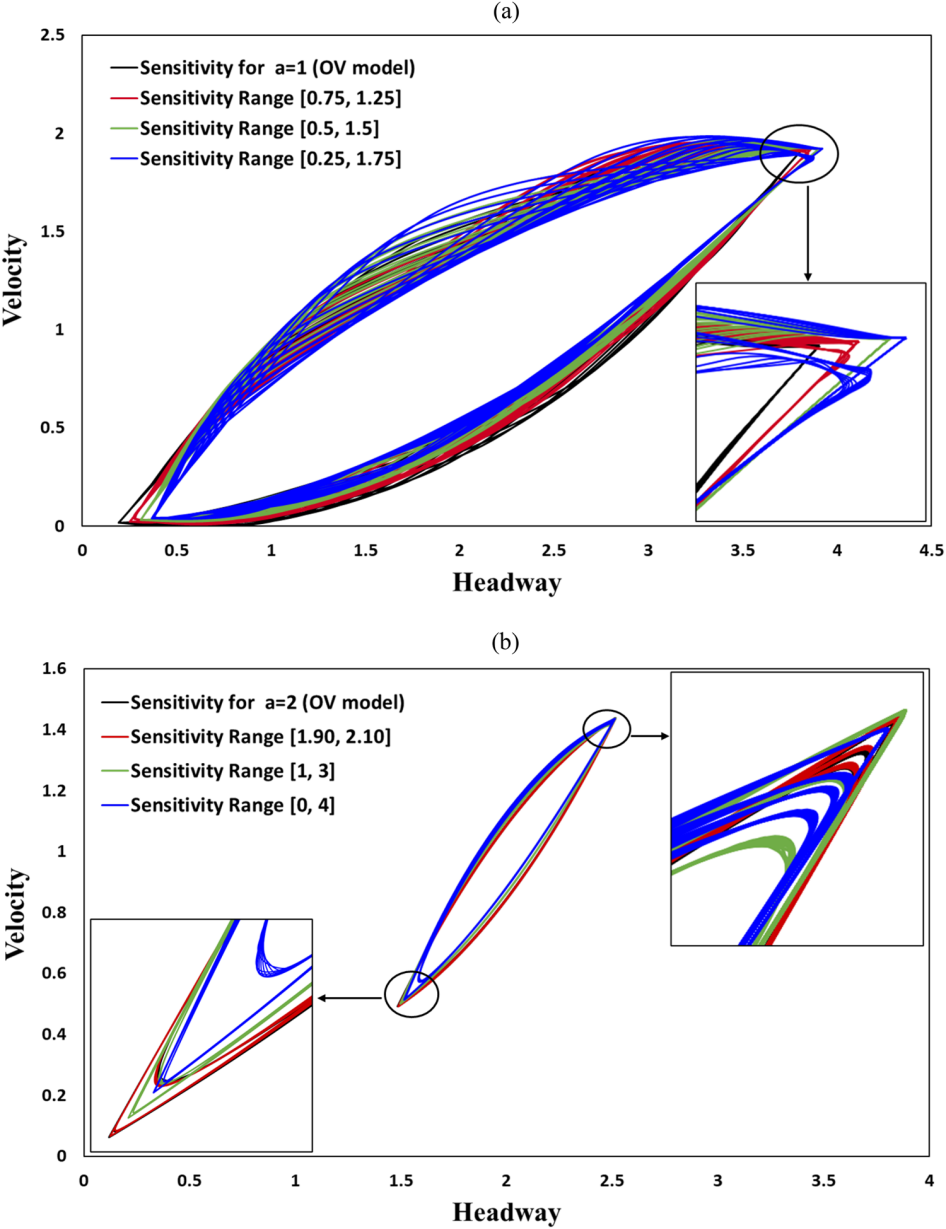
Hysteresis loops comparison analysis for the conventional OV for (**a**) $a = 1$, and (**b**) $a = 2$ and the HDDS models with several sensitivity ranges, while keeping the average of maximal and minimal sensitivities constant at (**a**) $a_{avg} = \frac{{a_{max} + a_{min} }}{2} = 1$, and (**b**) $a_{avg} = \frac{{a_{max} + a_{min} }}{2} = 2$. The insets show the detail of both the top and bottom vertices.

Figure 5a compares the different ranges of sensitivity in the HDDS model while keeping the average of $a_{min}$ and $a_{max}$ constant; the figure shows the results for the ranges [0.25, 1.75] (blue), [0.5, 1.5] (green), and [0.75,1.25] (red). For comparison, we additionally show the results of the OV model for $a = 1$ (black). Evidently, with the decrease in the dynamic range of sensitivities, the result approximates the OV result (black).

Figure 5b displays the same comparison, with the average of $a_{min}$ and $a_{max}$ increased from one to two. The same tendency as described above is observed. More importantly, we should note that the increase in the average of $a_{min}$ and $a_{max}$ (in the HDDS model) as well as *a* (in the OV model) renders the difference between those two models considerably smaller. This is also expected because the difference between the dynamic and static sensitivity models becomes less notable if the baseline sensitivity increases significantly.

**Figure 6:**
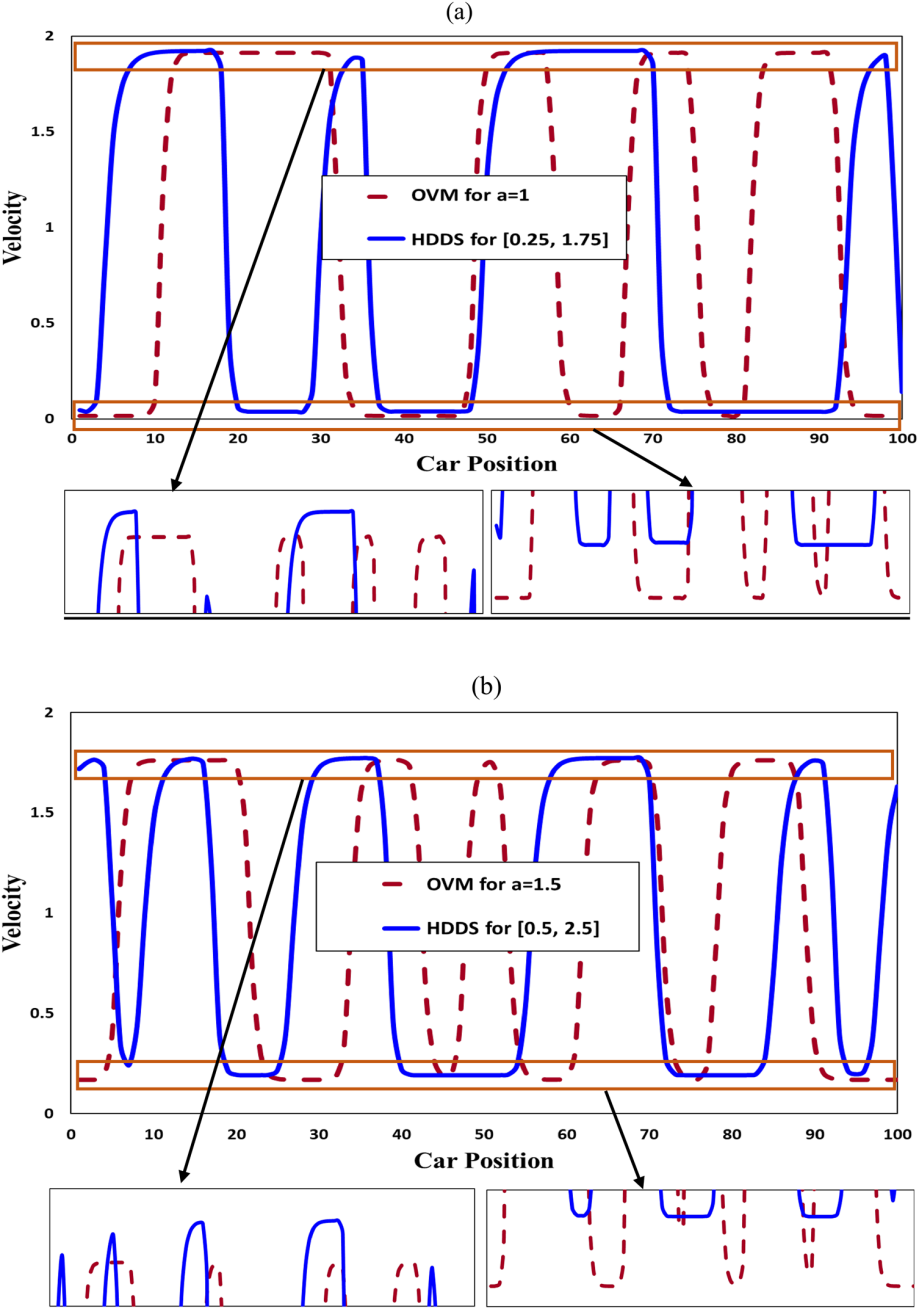
Comparison analysis of velocity profile of all vehicles at a particular time step, $t =$ 100,000, for the HDDS and conventional OV models. The panels show the results for (**a**) OV model $a = 1$ where HDDS model $a_{avg} = \frac{{a_{max} + a_{min} }}{2} = 1$ and (**b**) OV model $a = 1.5$ where HDDS model $a_{avg} = \frac{{a_{max} + a_{min} }}{2} = 1.5$.

Figure 6 shows the velocity profile of each of the 100 vehicles. As mentioned above, the HDDS model (blue) shows slightly larger maximal and minimal velocities than the OV model. It is worthwhile noting that the (positively) increasing slope of the HDDS model is significantly decreased just before reaching the peak velocity compared with that of the OV model. This is also due to the less sensitive manner in which the environment with large headway distances in the case of the HDDS model brings about inertial effects.

**Figure 7:**
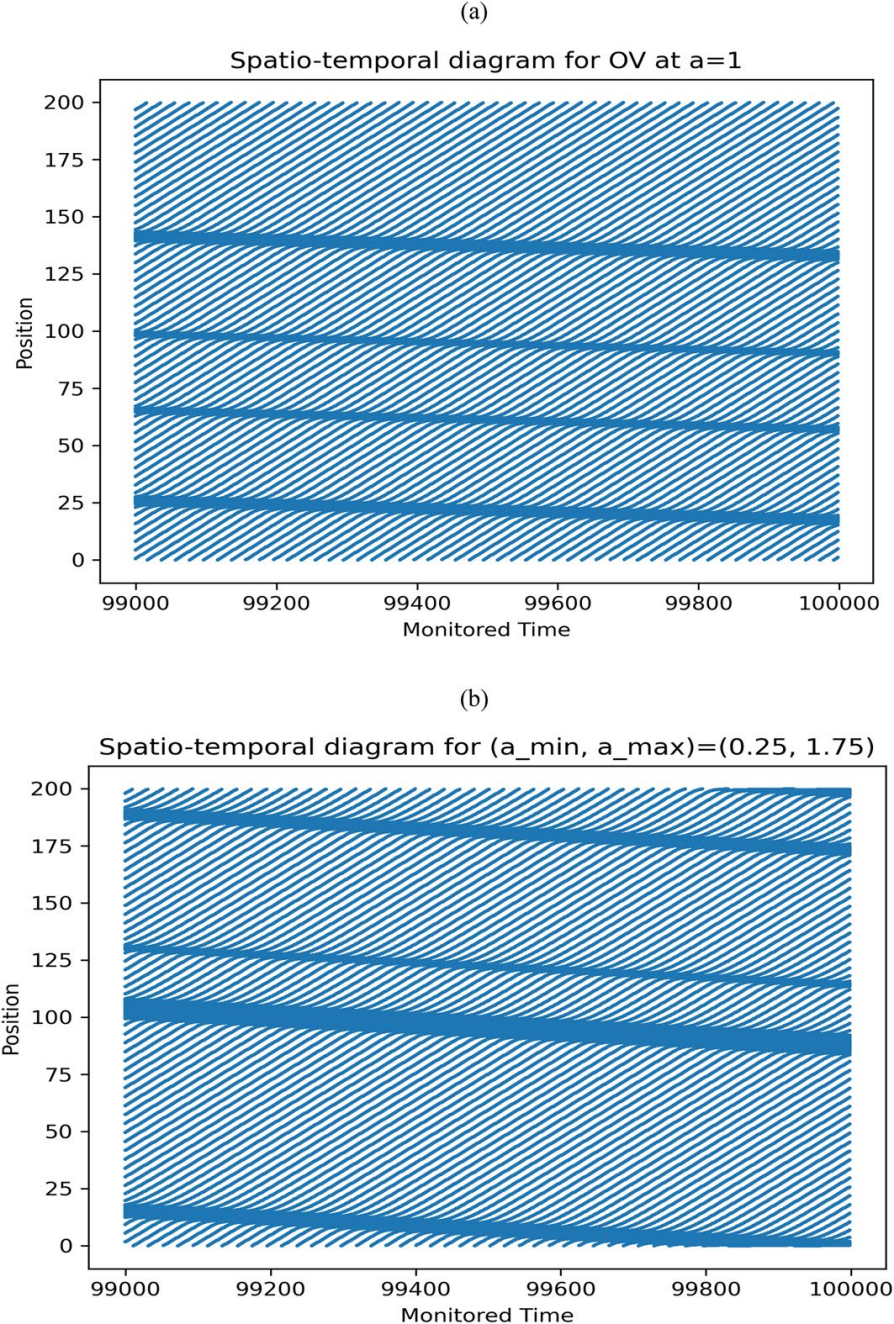
A comparison analysis for the spatiotemporal diagrams of the conventional OV model and proposed HDDS model.

A transparent scenario of the traffic flow behavior is depicted by the spatiotemporal diagram shown in Fig. 7. These panels show a comparative analysis of the flow field capacity for the conventional OV model and the proposed HDDS model. In this investigation, the flow field has been observed for 1000 time steps where the OV model was studied for $a = 1$ displayed in panel (a), and the developed HDDS model was examined for $\left( {a_{min} ,a_{max} } \right) = \left( {0.25, 1.75} \right)$ with keeping an average of $a_{min}$ and $a_{max}$ at 1 which is demonstrated in panel (b). It can be observed that both flow fields for OV and HDDS have the same number of jamming regions and that the average flow field velocity and traffic flux (number of vehicles passing per unit time) for these flow fields are comparable as shown in Fig. 7. But the main superiority of this improved HDDS traffic model is the implementation of a novel driver’s sensitivity that can trace out the driver’s attentiveness following the instantaneous traffic density.

## Conclusion

In this study, we have developed a new microscopic traffic flow model considering the effect of the driver's psychology (that is to say, heterogeneity of driver's sensitivity) on traffic flow fields. In The developed heterogeneous driver's sensitivity (HDDS) model, the driver's sensitivity function was defined as being dependent on his/her headway distance; where the variation of this parameter according to the headway distance is in contrast with that of the OV function.

We undertook a linear stability analysis, which demonstrated that the HDDS and OV models have similar tendencies with respect to the neutral stability condition. A series of numerical simulations reveal that the HDDS model shows different dynamic characteristics compared with the conventional OV model. Because of the higher sensitivity at smaller headway distances (i.e., in congested situations), the HDDS model allows higher minimum velocities and larger minimum headway distances than the OV model. By contrast, at the highest velocities with the largest headway distances, those two properties in the HDDS model take slightly higher values than those in the OV model. These two facts result from how the inertial effects of the HDDS model are implemented in the model's sensitivity. Even though the performance of the developed HDDS model is close to the conventional OV model. But the main advantage of this proposed HDDS traffic model is to introduce a new cognitive driver’s sensitivity in the traffic flow system that can express the mental behavior of the driver following the current headway distance, and this model solved a crucial drawback of the conventional traffic models and its followers that have been bearing over last two decade.

### Limitations and Future work

The current model introduces how time-variable, i.e., dynamic sensitivity, impact the fundamental features of a traffic flow, such as flow stability and efficiency. One limitation, which can be the focus of future work, is that individual diversity of the sensitivity is homogeneous. In reality, such a driver’s sensitivity should be modeled as a distribution. Furthermore, we want to extend this model from the microscopic to the macroscopic system.

## Supplementary Information

Supplementary Information 1.

Supplementary Information 2.

## Data Availability

All data generated and analyzed during this study are included in the supplementary information files. The simulation code of this model is also submitted as a supplementary file.
