# Supplementary material for: A dynamical traffic flow model for a cognitive drivers' sensitivity in Lagrangian scope
Source: Sci Rep. 2022 Oct 15;12:17341. doi: 10.1038/s41598-022-22412-9 (PMC9569355; doi:10.1038/s41598-022-22412-9)
Supplement: Supplementary file 2 — Supplementary Information 2. [file 41598_2022_22412_MOESM2_ESM.pdf]

```cpp
#include <iostream>
#include <fstream>
#include <stdio.h>
#include <math.h>
#include <vector>
#include <list>
#include <string.h>
#include <stdlib.h>
#include <ctype.h>
#include <sstream>
#include <string>
#include <cstdio>
#include <time.h>

using namespace std;




const double rho_step = 10.01;
const double  dt = 0.1;
const double rho_end = 0.25;
const double a_max = 0.5;
const double a_min = 0.1;
const double lamda = 0.5;
double rho_start = 0.25;
double rho = rho_start;
double L = 200.0;             // Lenth of the road
int N = 100;// (int)car_no;
double Time = 10000;          // Total running time of the simulation
double hc = 2.0;
double iters = Time / dt;   // total time steps
int Tsteps = (int)iters;

std::vector<std::vector<double>>x(N, std::vector<double>(Tsteps, 0));
std::vector<std::vector<double>>v(N, std::vector<double>(Tsteps, 0));
std::vector<std::vector<double>>v1(N, std::vector<double>(Tsteps, 0));
std::vector<std::vector<double>>fx(N, std::vector<double>(Tsteps, 0));
std::vector<std::vector<double>>fv(N, std::vector<double>(Tsteps, 0));
std::vector<std::vector<double>>dx(N, std::vector<double>(Tsteps, 0));
std::vector<double > A_V(Tsteps, 0);//, std::vector<double > q(Tsteps, 0);

inline double V(double dx) {

       return tanh(dx - hc) + tanh(hc);

}


inline double S(double dx) {

       return a_min + ((a_max - a_min) / (1 + exp(dx - hc)));

}

inline double rand_value(void) {
       double rr = (double)rand() / (double)RAND_MAX;
```

```
	//cout << "random number=" << rr << endl;
	return rr;

}

void Delx(vector<vector<double >>& x, vector<vector<double >>& dx, int& i) {
	for (int j = 0; j < N; j++) {

		if (j != N - 1) {
			dx[j][i] = x[j + 1][i] - x[j][i];
			//cout << dx << endl;
		}
		else {
			dx[j][i] = x[0][i] - x[N - 1][i];
			//cout << dx << endl;
		}
		if (dx[j][i] < -L * 0.5) dx[j][i] += L;

	}

}

void Headway(vector<vector<double >>& x, vector<vector<double>>& v, vector<vector<double
>>& fx, vector<vector<double>>& fv, int& i) {


	Delx(x, dx, i);
	for (int j = 0; j < N; j++) {

		fv[j][i] = S(dx[j][i]) * (V(dx[j][i]) - v[j][i]);


		//std::cout << k1 << endl;


	}

	for (int j = 0; j < N; j++) {
		fx[j][i] = v[j][i];

	}


}


void Model(vector<vector<double >>& x, vector<vector<double>>& v, int& i) {

	Headway(x, v, fx, fv, i);

	for (int j = 0; j < N; j++) {
		x[j][i + 1] = x[j][i] + (fx[j][i] * dt);
		v[j][i + 1] = v[j][i] + (fv[j][i] * dt);
		if (x[j][i + 1] > L) {
			x[j][i + 1] = x[j][i + 1] - L;
		}
	}
	/*
```

```
		for (int j = 0; j < N; j++) {
			if (j != N - 1) {
				v1[j][i] = v[j + 1][i];
			}
			else {
				v1[N - 1][i] = v[0][i];
			}
		}
		*/
}
/*
void Model(vector<vector<double >>& x, vector<vector<double>>& v, int& i) {

	Discretizes_equation(x, v, i);

	for (int j = 0; j < N; j++) {
		if (x[j][i] > L) {
			x[j][i] = x[j][i]-L;
		}
	}

}
*/
void initial(vector<vector<double>>& x, vector<vector<double>>& v) {
	double eps = 0.1;
	double dx = L / (double)N;
	double initial_velocity = V(dx);

	for (int j = 0; j < N; j++) {
		x[j][0] = L / (double)N * (double)j;// +eps * rand_value();
		v[j][0] = initial_velocity;

		//std::cout << x[i][j] << "," << v[i][j] << endl;
	}

	x[N / 2][0] += 0.05;
	x[(N / 2) + 1][0] -= 0.05;

	for (int j = 0; j < N; j++) {
		if (j != N - 1) {
			v1[j][0] = v[j + 1][0];
		}
		else {
			v1[N - 1][0] = v[0][0];
		}
	}

}


int main(void) {

	ostringstream file1;
	file1 << "diagram for a=(" <<a_min<<","<< a_max << ")" << ".csv";
	ofstream Data(file1.str().c_str(), ios_base::out | ios_base::trunc);
	Data << "Monitored Time,Position" << std::endl;

	initial(x, v);
```

```
	for (int i = 0; i < Tsteps - 1; i++) {
		Model(x, v, i);
	}

	for (int i = Tsteps-1001; i < Tsteps-1; i++) {
		for (int j = 0; j < N; j++) {

			std::cout << x[j][i] << endl;

			/*std::cout << x[j][i] / 2. << "," << dx[j][i] << "," << v[j][i] <<
endl;
			Data << x[j][i] / 2. << "," << dx[j][i] << "," << v[j][i] << endl;


			if ( i == Tsteps -1) {
				std::cout << x[j][i] << "," << v[j][i] << endl;
				Data << x[j][i] << "," << v[j][i] << endl;

			}*/



		}


	}
	return 0;
}
```
